# Effects and Safety of Press-Needle Therapy for Improving Visual Function and Eye Blood Circulation in Patients With Glaucoma With Controlled Intraocular Pressure: Study Protocol for a Multicenter Randomized Controlled Trial

**DOI:** 10.2196/67737

**Published:** 2025-04-01

**Authors:** Hongji Liu, Yan Dai, Ming Yu, Jian Zeng, Chao Wang, Sa Tan, Ming Xiong, Ran Zhang, Xuemeng Yu, Mingsong Shi, Xing Yan, Fengming Lai

**Affiliations:** 1 Department of Ophthalmology Mianyang Central Hospital, School of Medicine University of Electronic Science and Technology of China Mianyang, Sichuan China; 2 Department of Ophthalmology Mianyang Hospital of Traditional Chinese Medicine Mianyang, Sichuan China; 3 Department of Ophthalmology Mianyang Wanjiang Eye Hospital Mianyang, Sichuan China; 4 Department of Ultrasound, Mianyang Central Hospital School of Medicine University of Electronic Science and Technology of China Mianyang, Sichuan China; 5 NHC Key Laboratory of Nuclear Technology Medical Transformation Mianyang Central Hospital, School of Medicine University of Electronic Science and Technology of China Mianyang China

**Keywords:** press needle, press-needle therapy, needling technique, glaucoma, acupuncture, visual function, eye blood circulation, randomized controlled trial, traditional Chinese medecine

## Abstract

**Background:**

Glaucoma is the leading cause of irreversible blindness worldwide, causing continuous and progressively worsening damage to visual function, which leads to vision loss. Optic nerve protection is an important treatment for glaucoma with controlled intraocular pressure (GPCI), but to date, there is no universally accepted effective optic nerve protection agent. Acupuncture can protect the optic nerve by increasing blood flow to the eye. However, fear of pain or the limitations of treatment place and time lead to poor patient compliance. Press-needle therapy is a characteristic of traditional Chinese medicine (TCM) external treatment methods; its safety is high, the effect is fast and lasting, it is easy to conduct, and it has high patient compliance.

**Objective:**

The objective of the trial is to evaluate the safety and clinical efficacy of press-needle therapy and investigate whether it can improve visual function by regulating eye blood circulation in patients with GPCI.

**Methods:**

In total, 192 participants aged 18-75 years with GPCI from the Mianyang Central Hospital, the Mianyang Hospital of Traditional Chinese Medicine, and the Mianyang Wanjiang Eye Hospital will participate in this study. Participants will be allocated to 2 treatment groups (experimental and control groups) in a ratio of 1:1 and will undergo press-needle therapy and sham press-needle therapy, respectively, for the same 4-week period. Primary outcomes will include the best-corrected visual acuity (BCVA), optical coherence tomography angiography (OCTA), color Doppler flow imaging (CDFI), and visual field assessment results. Secondary outcomes will include the intraocular pressure (IOP) and traditional Chinese medicine (TCM) clinical symptom scales. The primary outcomes and safety assessments will be measured at baseline and 4 weeks thereafter, while the secondary outcomes will be measured at baseline and 1, 2, 3, and 4 weeks thereafter.

**Results:**

Recruitment and data collection began in February 2023. The final outcomes are expected in September 2025. As of October 2024, the project had recruited 220 eligible participants, of whom 192 (87.3%) will complete the study, exceeding initial projections for the study time frame. The remainder of the participants will provide the ability to explore cross-level interactions that could not be statistically powered at the outset. The strengths of the project include rigorous data collection, good retention rates, and high compliance rates.

**Conclusions:**

This study will provide data on the effects of press-needle therapy on visual function and ocular circulation in patients with GPCI, and these results will help demonstrate whether acupuncture can improve patients’ visual function by regulating ocular circulation, thus providing a clinical and theoretical basis for the wider application of acupuncture therapy in GPCI.

**Trial Registration:**

Chinese Clinical Trial Registry ChiCTR2300067862；https://tinyurl.com/mrxd58x9

**International Registered Report Identifier (IRRID):**

DERR1-10.2196/67737

## Introduction

Glaucoma is a leading cause of irreversible blindness worldwide [1]. Progressive degeneration of retinal ganglion cells (RGCs) and the optic nerve is the basic pathological characteristic of glaucoma [2]. Optic nerve protection is the focus of glaucoma treatment based on intraocular pressure (IOP) control. Neuroprotective targets include glutamate-induced neurotoxicity, nitric oxidase synthetase, neurotropins, calcium channel receptors, free radicals, vascular insufficiency, and the rho-kinase pathway [3]. Nevertheless, at present, there is no satisfactory neuroprotective agent for glaucoma.

Ocular blood flow disorder is a potential cause of visual field deterioration in patients with glaucoma [4]. Therefore, therapeutic options to protect the optic nerve in glaucoma by improving ocular blood flow have attracted increasing attention [3]. Acupuncture therapy has been used to treat glaucoma for thousands of years and has demonstrated favorable safety and convenience [5]. Acupuncture can modify glaucoma blood flow parameters to protect the optic nerve, such as increasing the flow of blood in the eye [6] and reducing anomalies of blood flow in the central artery of the retina [7]. Acupuncture treatments relay electrical signals via the dorsal root ganglion (DRG) and spinal cord, finally regulating ocular blood flow [8].

However, the fear of acupuncture pain or the limitations of the treatment place and time lead to poor patient compliance. Press-needle acupuncture is one kind of acupuncture that has a longer treatment duration and simpler operation with less pain [9]. The theoretical basis of press-needle therapy is the combination of acupuncture theory and skin meridian and acupoint theory. *Suwen: Treatise on the Dermis* states that “all twelve meridians are also part of the skin” and that “the skin is closely related to the veins. The skin is also part of the pulse.” The dermis is connected with the 12 meridians by qi and blood. Through the 12 meridians, the 12 dermises are in close contact with the 5 viscera, 6 internal organs, and the eye orifices, forming a coordinated and unified organic whole. Press-needle therapy has been widely used to treat glaucoma with controlled intraocular pressure (GPCI), but useful empirical research is insufficient for its popularization and application. The disadvantages of less rigorous designs, small sample sizes, and low quality have led to many traditional Chinese medicine (TCM) studies being insufficient to convince people of its value.

Hence, a randomized, sham-controlled trial will be conducted to assess the potential of press-needle therapy in improving visual function and eye blood circulation in patients with GPCI.

## Methods

### Research Objectives

The main objectives of this study are twofold: (1) to observe the clinical efficacy of press-needle therapy in patients with GPCI and provide feasible and affordable press-needle treatment for them and (2) to investigate whether press-needle therapy improves the visual function of patients with GPCI by regulating eye blood circulation.

### Trial Design and Setting

This multicenter, sham-controlled, blinded, randomized trial of press-needle therapy (Seilini Ltd) for GPCI will be executed in the Mianyang Central Hospital (Sichuan, China), the Mianyang Hospital of Traditional Chinese Medicine (Sichuan, China), and the Mianyang Wanjiang Eye Hospital (Sichuan, China). The study time schedule is presented in Table S1 in Multimedia Appendix 1.

### Ethical Considerations

The trial protocol was approved by the Science and Technology Department of Sichuan Province (approval no: 2021YJ0443), the National Natural Science Foundation of China for the Youth (approval no: 82305324), Mianyang Central Hospital Incubation Subjects (approval no: 2020FH07), and the Medical Ethics Committee of the Mianyang Central Hospital (approval no: S2021046 (02)). It has been registered with an identifier (ChiCTR2300067862) with ClinicalTrials. The study will be strictly conducted according to the principles of the Declaration of Helsinki, as well as Good Clinical Practice (GCP) guidelines. The trial will be performed in accordance with the SPIRIT (Standard Protocol Items: Recommendations for Interventional Trials) checklist [10]. Signed informed consent will be obtained from all patients or their legal guardians by specific researchers prior to beginning the trial. If there are changes to the eligibility criteria, outcomes, or analyses, a new version of the protocol will be submitted to the Medical Ethics Committee of the Mianyang Central Hospital for approval.

### Participants and Recruitment Strategy

In total, 192 patients with GPCI recruited from the Mianyang Central Hospital, (Sichuan, China), the Mianyang Hospital of Traditional Chinese Medicine (Sichuan, China), and the Mianyang Wanjiang Eye Hospital (Sichuan, China) will participate in this study. All patients will be screened by specialists and acupuncturists according to the diagnostic criteria in the *European Glaucoma Society Terminology and Guidelines for Glaucoma* [11]. The TCM syndrome of kidney deficiency and blood stasis will be diagnosed based on guidelines delineated in the *Clinical Research Guideline of New Investigational Drugs in Traditional Chinese Medicine* [12].

### Inclusion and Exclusion Criteria

Participants who meet the following inclusion criteria will be eligible for the study:

- Diagnosed with primary open-angle glaucoma (POAG) [13]: open anterior chamber angle, pathologically high intraocular pressure (HIOP), with a peak IOP exceeding 21 mmHg (1 mmHg=0.133 kPa) within a 24-hour period, indicative of glaucomatous neuropathy (retinal nerve fiber layer defect [RNFLD] or optic disc changes) or visual field loss or both. Other causes resulting in HIOP were excluded for this diagnosis. Note that only 1 eye from each patient will be selected for examination.
- Having a visual field defect that can be assessed through standard automated perimetry with a reliability factor of less than 15%, as well as an IOP below 18 mmHg, at least 3 months after undergoing antiglaucoma surgery.
- Exhibiting a Chinese syndrome pattern associated with kidney deficiency and blood stasis.
- Refraining from the use of any other optic nerve protective agents, except for methylcobalamin, for a minimum of 2 months.
- Having a spherical refractive error ranging between +3.00 and –6.00 diopters, a fundus clearly visible without the need for pupil dilation, and a BCVA equal to or greater than 0.3.
- Within the age bracket of 18-75 years.
- In a conscious state and capable of cooperating with the examination and treatment process.
- Displaying a willingness to actively participate in the study and provide informed consent.

Participants who have any of the following conditions will not be eligible to participate:

- Poorly controlled IOP (≤7 mmHg or >18 mmHg), accompanied by a thin filtering cystic bleb or bleb leakage.
- Glaucoma that is not open-angle glaucoma.
- A Chinese syndrome pattern that does not meet the criteria of kidney deficiency and blood stasis.
- Other conditions, such as macular degeneration, cataract, proliferative diabetic retinopathy, retinal vascular occlusion, or any other diseases that may cause visual field loss.
- Pregnancy or lactation.
- Abnormal primary liver or kidney function or serious systemic conditions, such as heart disease, primary hypertension, diabetes mellitus, or peptic ulcer disease.
- A history of allergic reactions to the ingredients of press-needle therapy or mecobalamin tablets.

### TCM Syndrome Differentiation

The TCM syndrome of kidney deficiency and blood stasis will be based on guidelines delineated in the *Clinical Research Guideline of New Investigational Drugs in Traditional Chinese Medicine* [12]: primary signs and symptoms include blurry visual acuity, narrow visual field, and eye distension, while secondary signs and symptoms include dry eye, limp aching lumbus, and knees.

**Figure 1:**
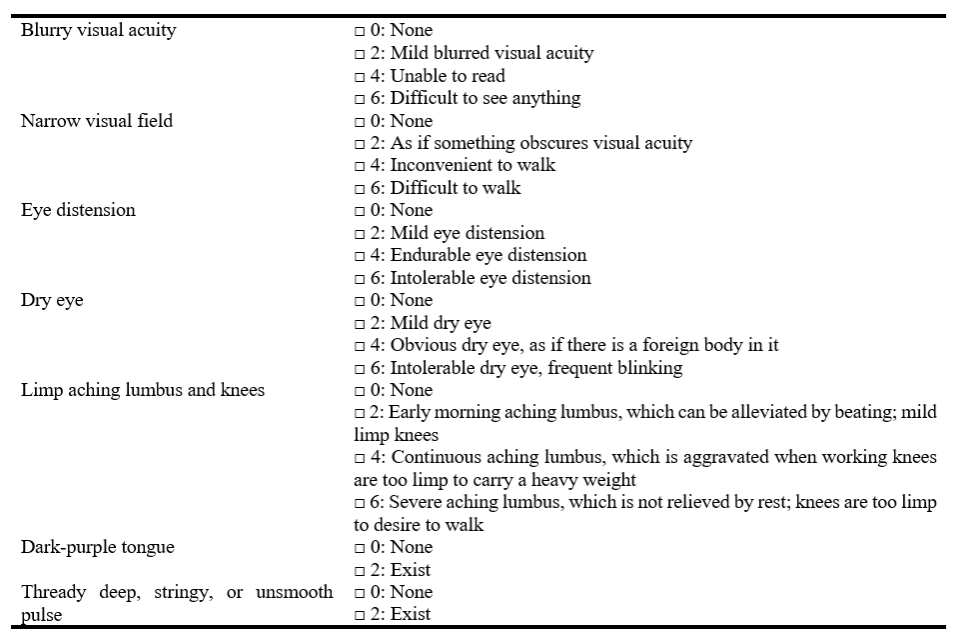
TCM Symptom Score Scale. TCM: traditional Chinese medicine.

The conditions for a diagnosis of kidney deficiency and blood stasis are the presence of at least 2 or more primary signs/symptoms and at least 2 or more secondary signs/symptoms. The necessary condition is a dark-purple tongue and a thready deep, stringy, or unsmooth pulse. The investigators must receive training in standard operating procedures (SOPs) for examining TCM symptoms. They will then conduct a symptom assessment survey using the TCM Symptom Score Scale (Figure 1) for each participant.

### Recruitment Procedure

Potential participants with GPCI were recruited from the Mianyang Central Hospital (Sichuan, China), the Mianyang Hospital of Traditional Chinese Medicine (Sichuan, China), and the Mianyang Wanjiang Eye Hospital (Sichuan, China). We used the internet, hospital announcements, and posters to provide a hotline calling for potential volunteers. If patients were interested in our study, researchers informed them in detail of the purpose and content of the research and the benefits and risks of participating.

After screening, participants meeting the inclusion criteria will be enrolled. Frequent follow-up phone calls are an important aspect of adherence monitoring. Every participant will have the right to withdraw from the trial at any time. Furthermore, it will be obligatory for all subjects to sign an informed consent document prior to commencement of the trial.

### Randomization and Allocation

Baseline evaluation will be conducted for eligible subjects. All eligible patients will be stratified according to early, moderate, and advanced stages of glaucoma [14]. Patients with early glaucoma exhibit early glaucomatous visual field impairment, as characterized by a mean deviation (MD) of >–6 dB. Conversely, patients with moderate glaucoma present a moderate glaucomatous visual field loss, defined as MD≤–6 to ≥–12 dB. Patients with advanced glaucoma experience advanced glaucomatous visual field loss, defined as MD<–12 dB. SAS software will be used to automatically generate a set of random numbers ranging from 001 to 192. These numbers will be further classified into 3 ranges based on the disease stage. The early stage will incorporate random numbers from 001 to 064, the moderate stage will encompass numbers from 065 to 128, and the advanced stage will comprise numbers from 129 to 192. Following stratification, the participants will be assigned randomly to either an experimental group or a control group in a 1:1 ratio. Random numbers will be inserted into identical envelopes of equal dimensions, which will then be sealed to ensure concealment of group allocation and minimize the risk of selection bias. The random sequence allocation will be preserved and withheld from the investigators, statisticians, and outcome assessors to avoid detection bias.

#### Blinding

The researcher assistants, outcome assessors, statisticians, and subjects will be blinded to the group assignment. All participants will undergo press-needle therapy while lying on a bed in a tranquil clinic setting. For the control group, a nonpenetrating sham apparatus, similar in appearance to acupuncture needles, will be used to achieve blinding. Moreover, every participant will be asked about their experience subsequent to the acupuncture session to evaluate the efficacy of blinding. Unblinding of researchers is permitted only in exceptional circumstances, such as when there is a strong need to know about the actual treatment in order to manage the subject appropriately.

### Interventions

#### Experimental Group

Acupoints will be selected based on acupuncture combined with clinical experience [15]: main acupuncture points (BL2 Cuanzhu, TE23 Sizhukong, EX-HN5 Taiyang, and ST2 Sibai) and matching points (PC6 Neiguan, SP10 Xuehai, BL17 Geshu, BL18 Ganshu, BL23 Shenshu, SP6 Sanyinjiao, and K13 Taixi) (Figure 1). These points can exert a concerted effect to nourish the liver and kidneys, promote blood circulation, and dredge collaterals. Press-needle treatment will be performed by acupuncturists with more than 3 years of clinical experience. The acupuncturist will disinfect the skin around the acupoints (on the same side of the affected eye) with povidone iodine when the subject lies down on the treatment bed in a supine position. Disposable sterile press needles (0.2 mm in diameter and 0.6 mm in length; Seirin Corporation) will then be inserted into the right acupoints (Figure 1). The needles will be pressed so that they can adhere and be tied, the peelable paper will be removed, and the adhesive tape will be pressed down to ensure a secure fit. The needles will be left in place for 2 days, and participants will be asked to press the needles themselves 3-4 times/day, every 4 hours, for 1 minute each time to achieve deqi sensations, including soreness, numbness, heaviness, fullness, and aching.

#### Control Group

Sham press needles are the same as press needles but lack the needle element [9]. The procedures and manipulations carried out in the control group will be indistinguishable from those implemented in the experimental group. Subjects will be treated individually in a secluded room to prevent any disruptions from family members or other individuals.

#### Criteria for Discontinuing or Modifying Allocated Interventions

Participants will have the freedom to withdraw from the clinical study and opt for alternative treatments (eg, citicoline sodium tablets) if they believe their condition will not improve. Reasons for discontinuing treatment may include, but are not limited to, the following: (1) the pregnancy, (2) severe side effects or complications, (3) taking other medications during the study, (4) failure to comply with the study protocol, and (5) request for withdrawal for other reasons.

### Outcome Measures

#### Primary Outcome Measures

The primary outcomes will enable us to evaluate improvements in visual function, retinal blood circulation, and retrobulbar blood vessel circulation due to press-needle therapy. The outcomes will be as follows:

- The mean change in the BCVA will be measured at baseline and 4 weeks thereafter.
- The mean change in the values of the pattern standard deviation (PSD), mean defect, and square root of loss variance (sLV) will be determined using an Octopus 900 perimeter at baseline and 4 weeks thereafter.
- The whole-image vessel density (wiVD), radial peripapillary capillary vessel density (RPCVD), superficial macula vascular density (smVD), parafoveal vessel density (paraVD), perifoveal vessel density (periVD), macular ganglion cell complex (mGCC), and peripapillary retinal nerve fiber layer (pRNFL) will be determined using optical coherence tomography angiography (OCTA) at baseline and 4 weeks thereafter.
- The ophthalmic artery (OA), central retinal artery (CRA), short posterior ciliary artery (SPCA), peak systolic velocity (PSV), end diastolic velocity (EDV), and resistance index (RI) will be determined using CDFI at baseline and 4 weeks thereafter.

#### Secondary Outcome Measures

The following secondary outcomes will help us determine whether press-needle therapy improves TCM clinical symptoms and changes in the IOP:

- The mean change in the IOP in the study eye will be determined uisng a TX-20P full autotonometer at baseline and at 1, 2, 3, and 4 weeks thereafter.
- Changes in TCM symptoms will be assessed by the investigators trained in SOPs according to the TCM Symptom Score Scale (Table S1 in Multimedia Appendix 1) at baseline and at 1, 2, 3, and 4 weeks thereafter.

### Safety Evaluation and Adverse Events

Safety assessments using 3 routine tests (blood, urine, and stool), electrocardiography, liver function tests (alanine aminotransferase [ALT], aspartate aminotransferase [AST], alkaline phosphatase [ALP], gamma-glutamyl transferase [GGT], and serum total bilirubin [TBIL]) and kidney function tests (blood urea nitrogen [BUN], serum creatinine [SCr], uric acid [UA], and β 2-microglobulin) will be measured at baseline and 4 weeks thereafter.

A research assistant will monitor and record data regarding adverse events (AEs) associated with interventions, which may include infection, hematoma, severe pain, needle breakage, bleeding, heart palpitations, fainting, and headaches, in case report forms (CRFs). Severe AEs will be managed by emergency medicine physicians or acupuncturists. Additionally, any significant AEs will be reported to the Ethics Committee of the Mianyang Central Hospital.

### Data Collection and Quality Control

Thorough recording of all data will be performed on CRFs subsequent to in-person visits or telephonic communications with participants. The same examiner will assume responsibility for conducting the examination of every patient during each assessment. Baseline measurements, demographics, medical history, and prior medications will only be gathered during the initial visit. Primary outcomes, secondary outcomes, and safety indicators will be gathered at baseline and 4 weeks thereafter. Following adequate training and successful completion of a test, 2 data entry clerks will thoroughly review the CRFs and subsequently enter the collected data independently. Participants’ personal information will be kept confidential the same way as their medical histories in the hospitals before, during, and after the trial. The absence of a data monitoring committee in this study is justified by the anticipated low incidence of AEs and the limited participant numbers at each center. The Epidata 3.1 statistical application will be used for data entry. Subsequently, the collected data will be transferred to IBM SPSS 22.0 statistical software for the purpose of analysis.

### Sample Size Calculation

The sample size calculation was based on previous studies. The basis of optic nerve protection effectiveness is 70% [16,17]. We hypothesized an 80% effective rate of the basis of optic nerve protection + press needles for GPCI in a larger sample size. According to the sample size of the estimation formula [18], α=.05 and β=0.10 in a 2-sided test, *P*_1_=.67 and *P*_2_=.87, 2-sided u_α/2_=u_0.05/2_=1.96, and 1-tailed u_β_=u_0.1_=1.282. We substituted these values into the following formula:




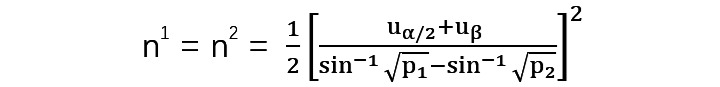




This resulted in n^1^=n^2^=79.02, that is, approximately 80 cases each in the experimental and the control group, or 160 cases in both groups. To account for a 20% dropout rate, the calculation 160 + (160 × 20%) = 192 suggested that 192 patients be recruited.

#### Quality Control

To guarantee the quality of the proposed experiment, a unified and standardized training program will be conducted for the researchers in each role prior to commencement of the study. Subsequently, the researchers will be subjected to a proficiency assessment, and only those deemed competent will be permitted to participate in the study. Furthermore, to enhance participant compliance, regular health education activities will be conducted, humane care will be enhanced, and a long-term follow-up plan will be developed. Finally, in accordance with the requirements set forth in the Specifications for Quality Control and Quality Assurance of Clinical Research in Chinese Medicine [19], a 4-level quality monitoring system will be established to monitor the clinically collected data on a regular monthly basis, thereby ensuring the authenticity and reliability of the study results.

### Statistical Analysis

Statistical analysis will be conducted independently by statisticians. The data will be presented as means (SDs). IBM SPSS 22.0 statistical software will be used. All statistical tests will be 2-sided tests, and the limit for statistical significance will be set at *P*<.05. The chi-square test will be performed for categorical variables, the independent Student *t* test will be used for measurement data, and the rank-sum test will be used for grade data. To evaluate the covariate balance, baseline characteristics will be summarized and compared between the experimental group and the control group using simple descriptive statistics.

Due to the expected low number of missing data, we will deal with missing data by transferring the last data item to the final dataset. This participant's data will be invalidated in the full analysis set (FAS), and this case will not be included in the per protocol set (PPS). Finally, the statistical significance of the FAS and PPS will be analyzed. A consistent statistical significance will indicate a significant difference.

## Results

**Figure 2:**
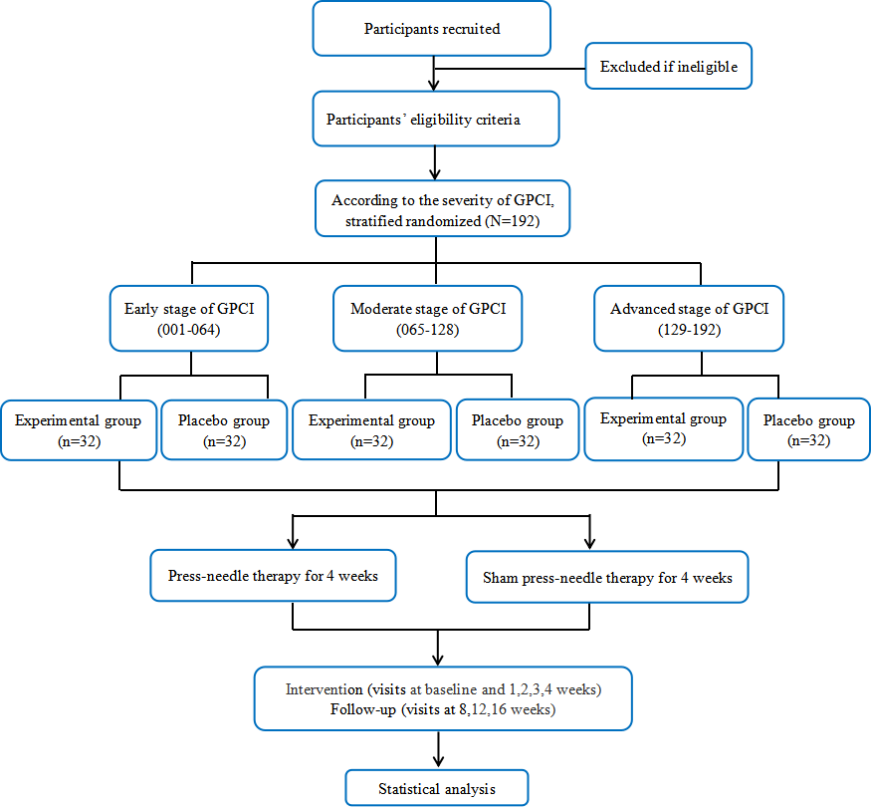
Flowchart of the clinical trial on patients with GPCI. GPCI: glaucoma with controlled intraocular pressure.

The study flowchart is shown in Figure 2. The figure also presents an outline of the case distribution across each center, as well as comprehensive outline of precise measurements and designated data collection time points. Recruitment was conducted from February 2023 to March 2024. The first participant was enrolled in February 2023. As of October 2024, the project had recruited 220 eligible participants. At the time of submission, there were 192 (87.3%) participants, who will complete the study. The recruitment process is expected to continue until September 2025. The data will be further processed and analyzed in October 2025.

## Discussion

### Overview

Acupuncture is a major treatment in TCM, and studies have shown that acupuncture can protect the optic nerve in patients with glaucoma [20]. The press needle is a new kind of acupuncture device that can create a more lasting stimulus and simpler operation, while resulting in less pain, with the therapeutic effect of cumulative treatment characteristics. At present, press-needle therapy is widely used in various clinical diseases and has achieved satisfactory therapeutic effects [9,21]. Acupuncture is effective in protecting visual function in glaucoma [22]. Press-needle embedding can improve the deteriorating vision and shrinking visual field of patients with optic nerve atrophy and increase clinical efficacy [23]. Abnormal blood flow is an important factor in optic nerve damage in glaucoma [24], and acupuncture can modify the blood flow parameters in glaucoma [7]. Clinically, we have widely used press-needle therapy for glaucoma visual function protection and achieved good clinical efficacy, and we have further found that press-needle therapy can improve ocular circulation in patients with glaucoma. However, the quality of evidence supporting the use of press-needle therapy for GPCI is limited due to methodological limitations. Therefore, it is necessary to explore the effect of press-needle therapy on visual function and eye blood circulation in patients with GPCI. The double-blind method reduces the differential assessment of outcome events and improves subject adherence. Previous studies have shown that press needles and sham press needles (lacking the needle element) enable effective double-blind settings for acupuncture clinical studies [25]. Therefore, sham press needles were chosen as the control intervention for this study.

Due to the unique nature of glaucoma, patients experience progressive optic nerve damage as the disease progresses. To avoid violating medical ethics and reduce the rate of patient detachment, methylcobalamin has been chosen as the basic optic neuroprotective drug for the experimental and control groups in this study. In addition, to observe the effect of press-needle therapy on GPCI of different disease severities, participants will be classified as early, moderate, and advanced based on their visual field defects.

This study is designed as a double-blind, placebo-controlled trial. To guarantee the quality of the study, its design and execution will be strictly performed with proper quality control. A training session for each center will be held to explain the detailed study protocol, the diagnosis of the TCM syndrome pattern, and the SOPs. The same investigator will be responsible for the examination of each patient at different times. All indicators will be assessed by independent assessors. The principal investigators, statisticians, and outcome assessors will be blinded to the treatment assignments until the database is locked.

### Innovations and Limitations

This is the inaugural clinical trial protocol devised by our team to investigate the potential mechanisms of acupuncture for patients diagnosed with GPCI. We have selected eye blood circulation as the entry point for this study. The trial will be conducted in accordance with rigorous methodology. For example, patients with GPCI will be randomly assigned to 2 groups. For the control group, a nonpenetrating sham apparatus, similar in appearance to acupuncture needles, will be used to achieve blinding. Furthermore, each participant will be requested to provide feedback on their experience following the acupuncture session, with a view to evaluating the efficacy of the blinding process. The data will be subjected to statistical analysis in a blinded trial, with the intervener, efficacy assessor, and statistician all acting independently. However, it is not possible to eliminate gender bias in the randomization process. Consequently, to guarantee the reliability of the data, it is necessary to expand the sample size further.

### Conclusion

In conclusion, the purpose of this research is to verify that press-needle therapy can effectively improve visual function and eye blood circulation in patients with GPCI. The outcome of this study will provide evidence-based data for the use of press-needle therapy to treat GPCI accompanied by liver-kidney deficiency, blood stasis, and fluid retention syndrome, thereby providing a new avenue for the treatment of GPCI.

## Appendix

Multimedia Appendix 1 Measurement items and points of data capture.

## Abbreviations

AE: adverse event
BCVA: best-corrected visual acuity
CDFI: color Doppler flow imaging
CRF: case report form
FAS: full analysis set
GPCI: glaucoma with controlled intraocular pressure
HIOP: high intraocular pressure
IOP: intraocular pressure
MD: mean deviation
OCTA: optical coherence tomography angiography
PPS: per protocol set
RGC: retinal ganglion cell
SOP: standard operating procedure
TCM: traditional Chinese medicine
