# Supplementary material for: Effects and Safety of Press-Needle Therapy for Improving Visual Function and Eye Blood Circulation in Patients With Glaucoma With Controlled Intraocular Pressure: Study Protocol for a Multicenter Randomized Controlled Trial
Source: JMIR Res Protoc. 2025 Apr 1;14:e67737. doi: 10.2196/67737 (PMC12000785; doi:10.2196/67737)
Supplement: Multimedia Appendix 1 [file resprot_v14i1e67737_app1.docx]

**Table S1.** Measurement items and points of data capture.

| Measurement items | | Baseline | Treatment period (weeks) | | | | Follow-up period (weeks) | | |
| --- | --- | --- | --- | --- | --- | --- | --- | --- | --- |
|  | |  | 1 | 2 | 3 | 4 | 8 | 12 | 16 |
|  | |  |  |  |  |  |  |  |  |
| **Enrollment** | | | | | | | | | |
|  | Eligibility screen | × |  |  |  |  |  |  |  |
|  | Informed consent | × |  |  |  |  |  |  |  |
|  | Medical history | × |  |  |  |  |  |  |  |
|  | Randomization | × |  |  |  |  |  |  |  |
| **Intervention** | | | | | | | | | |
|  | Experimental group |  |  |  |  |  |  |  |  |
|  | Sham press-needle group |  |  |  |  |  |  |  |  |
| **Assessment: primary outcomes** | | | | | | | | | |
|  | BCVA^a^ | × |  |  |  | × | × | × | × |
|  | OCTA^b^ | × |  |  |  | × |  |  |  |
|  | CDFI^c^ | × |  |  |  | × |  |  |  |
|  | Visual field | × |  |  |  | × |  |  |  |
| **Assessment: secondary outcomes** | | | | | | | | | |
|  | IOP^d^ | × | × | × | × | × | × | × | × |
|  | TCM^e^ clinical symptom scale | × | × | × | × | × | × | × | × |
| **Assessment: safety outcomes** | | | | | | | | | |
|  | Blood routine test | × |  |  |  |  |  |  |  |
|  | Urine routine test | × |  |  |  |  |  |  |  |
|  | Stool routine test | × |  |  |  |  |  |  |  |
|  | Liver function tests | × |  |  |  |  |  |  |  |
|  | Kidney function tests | × |  |  |  |  |  |  |  |
|  | Electrocardiogram exam | × |  |  |  |  |  |  |  |
|  | Adverse events |  | × | × | × | × | × | × | × |

^a^BCVA: best-corrected visual acuity.

^b^OCTA: optical coherence tomography angiography.

^c^CDFI: color Doppler flow imaging.

^d^IOP: intraocular pressure.

^e^TCM: traditional Chinese medicine.
